# Streptococcus mitis Abscesses Mimicking Liver Metastases

**DOI:** 10.7759/cureus.8852

**Published:** 2020-06-26

**Authors:** Mohamed Elnaggar, Sumaiya Mahboob, Bryce D Beutler, Ahmed Hanfy, Omar Canaday

**Affiliations:** 1 Internal Medicine, University of Nevada, Reno School of Medicine, Reno, USA

**Keywords:** abscesses, liver metastases, pyogenic abscess, streptococcus mitis

## Abstract

Streptococcus mitis (S. mitis) is a commensal bacterial species that commonly colonizes the oropharynx and gastrointestinal tract. It is seldom reported as a human pathogen. However, immunocompromised individuals may be at risk of infection. We describe a 62-year-old male with prostate cancer who presented with multiple S. mitis abscesses masquerading as metastases. In addition, we discuss the differential diagnosis and treatment options for this rare opportunistic infection.

## Introduction

Streptococcus mitis (S. mitis, previously known as Streptococcus mitior) is a mesophilic, facultatively anaerobic, gram-positive coccus. A member of the viridans group streptococci (VGS), S. mitis is most commonly found in the throat, nasopharynx, and oral cavity. It usually considered to have low virulence and pathogenicity. However, S. mitis endocarditis and meningitis have been reported [1,2]. 

In the United States, liver abscesses are most commonly polymicrobial and attributed to Escherichia coli and Klebsiella pneumoniae [3]. Entamoeba histolytica and Candida species account for the majority of non-bacterial abscesses [4,5]. We describe a patient with prostate cancer who presented with multiple liver lesions that were later revealed to be pyogenic abscesses caused by S. mitis.

## Case presentation

A 62-year-old male with stage III prostate cancer undergoing treatment with leuprolide and radiation therapy presented with a one-week history of anorexia, night sweats, subjective fevers, and abdominal pain.

The patient was afebrile and hemodynamically stable in the emergency department. Physical examination was significant only for right-sided abdominal tenderness. Laboratory studies showed a mild leukocytosis (white blood cell count: 13.3 K/µL; reference range: 4.5-11.0 K/µL) with bandemia, anemia (hemoglobin: 8.5 g/dL; reference range [male]: 14.0-18.0 g/dL), and elevated alkaline phosphatase (alkaline phosphatase: 276 U/L; reference range: 20-140 U/L). Prostate-specific antigen was <0.01 ng/mL (reference range: 0.0-4.0 ng/mL). Bilirubin, alanine aminotransferase, and aspartate aminotransferase were within normal limits. Renal function studies were unremarkable. Urinalysis was negative for leukocyte esterase and nitrites. Correlation of the clinical history and physical examination findings was worrisome for an occult infection, prompting emergency department providers to obtain additional imaging.

**Figure 1:**
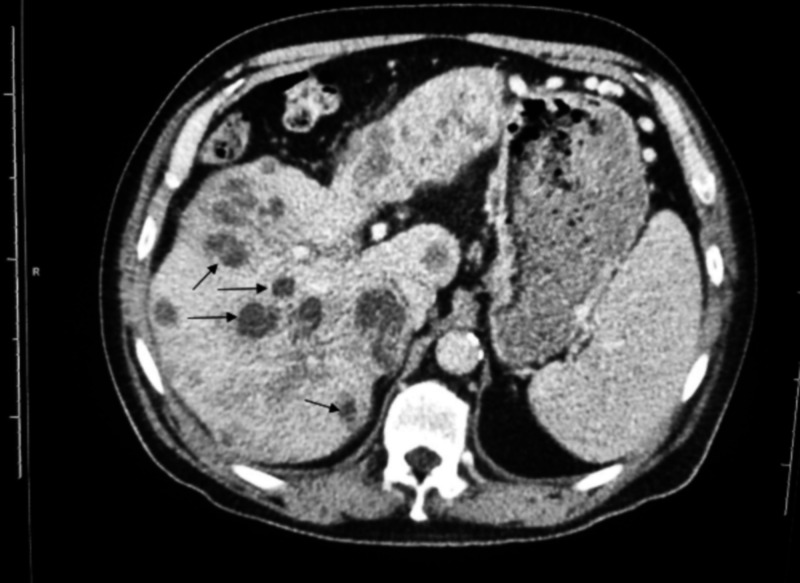
Axial view CT scan of the abdomen and pelvis with contrast shows numerous peripherally enhancing, centrally hypoattenuating masses in liver segments II, III, and IV.

An abdominal ultrasound demonstrated several poorly demarcated hypoechoic masses affecting the liver. A CT scan of the abdomen and pelvis with contrast was obtained to further characterize the lesions; this showed numerous peripherally enhancing, centrally hypoattenuating masses in liver segments II, III, and IV (Figure 1). All lesions were new as compared to a CT scan from two months earlier.

The differential diagnosis included liver abscesses and cystic metastases. Blood, sputum, stool, and urine cultures were acquired, and the patient was started on intravenous ceftriaxone and metronidazole. A final needle biopsy of a lesion was obtained for pathologic examination. 

**Figure 2:**
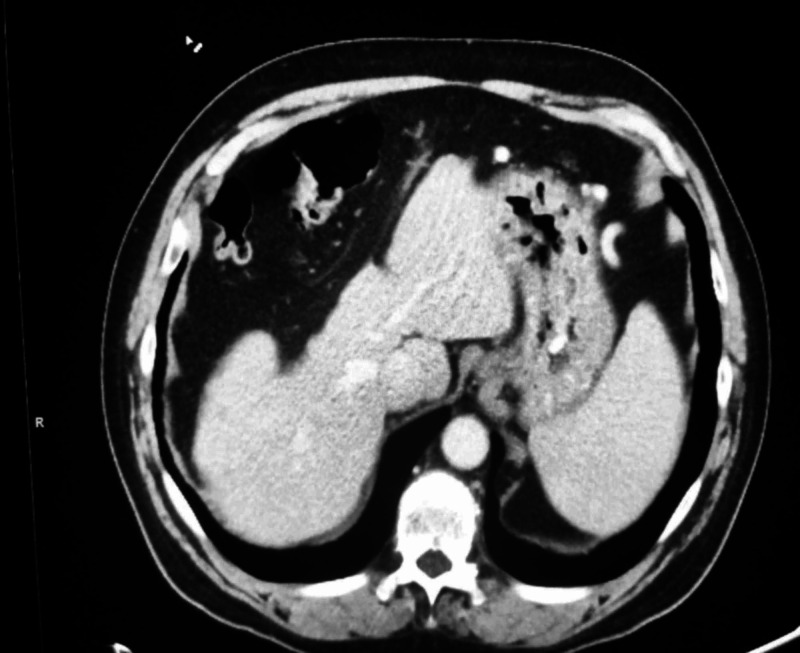
Axial view of a CT scan of the abdomen and pelvis with contrast demonstrates complete resolution of the previously observed Streptococcus mitis abscesses.

Tissue cultures grew S. mitis. Infectious disease was consulted and recommended intravenous ceftriaxone and metronidazole. The patient’s symptoms gradually began to improve, and he was discharged in a stable condition after a two-week hospital stay. Daily infusions of intravenous ceftriaxone was continued on an outpatient basis for a duration of six weeks. The patient continued to follow up with the infectious disease clinic. Repeat CT scan of the abdomen and pelvis with contrast obtained one month after completion of antibiotic therapy revealed resolution of the abscesses (Figure 2).

## Discussion

S. mitis is a rare but important opportunistic pathogen. In the setting of cancer, S. mitis abscesses may appear similar to metastases on imaging. Therefore, careful correlation of clinical history, physical examination, and laboratory and imaging findings is essential in order to establish a diagnosis. In our patient, pyogenic abscesses were favored over metastases for the following reasons: (1) the subacute onset of subjective fevers and abdominal pain is consistent with infection; (2) the liver represents an uncommon site for prostate cancer metastases; (4) prostate-specific antigen remained within normal limits; and (4) imaging studies obtained two months prior to presentation revealed no hepatic lesions.

S. mitis is a commensal organism rarely known to cause infection. To the best of our knowledge, only two other cases of S. mitis abscesses have been described in the medical literature [6,7]. Our patient was unique in that he had no history of immunosuppression. Previous studies of S. mitis bacteremia have shown that this opportunistic pathogen is usually susceptible to ceftriaxone; tetracycline represents an alternative therapy for individuals who are unable to tolerate cephalosporins [8]. In the setting of S. mitis abscesses, prolonged therapy may be required.

## Conclusions

Pyogenic abscesses are typically polymicrobial. However, albeit rarely, healthy patients may be susceptible to infection with atypical organisms, including S. mitis. The imaging findings of S. mitis abscesses are virtually indistinguishable from those of metastases. Careful clinical correlation is therefore required to establish a diagnosis and administer the appropriate treatment.
